# A new species of wasp-mimicking clearwing moth from Peninsular Malaysia with DNA barcode and behavioural notes (Lepidoptera, Sesiidae)

**DOI:** 10.3897/zookeys.692.13587

**Published:** 2017-08-21

**Authors:** Marta Agnieszka Skowron Volponi, Paolo Volponi

**Affiliations:** 1 Department of Molecular Biology, Faculty of Biology, University of Gdansk, Wita Stwosza 59, 80-308 Gdansk, Poland; 2 ClearWing Foundation for Biodiversity, ul. Podczaszyńskiego 11/15 m 23A, 01-866 Warsaw, Poland

**Keywords:** Sesiidae, clearwing moth, *Pyrophleps ellawi*, mimicry, mud-puddling, behaviour, Malaysia

## Abstract

A new species of clearwing moth, *Pyrophleps ellawi* Skowron Volponi, **sp. n.**, is described from Peninsular Malaysia. Information on the habitat, time and conditions of occurrence, flight and mud-puddling behaviour, functional morphology, and DNA barcode are also provided. Photographs and a supplementary video from the wild demonstrate the postures and behaviour of this species of *Pyrophleps*, whose remaining members were described only on the basis of pinned specimens. This is the first record of this genus in Peninsular Malaysia.

## Introduction

Until now the genus *Pyrophleps* Arita & Gorbunov, 2000 comprised seven species. Three were described more than one hundred years ago, namely *Adixoa cruentata* Swinhoe, 1896, *Aegeria ruficrista* Rothschild, 1912 and *Aschistophleps haematochrodes* Le Cerf, 1912. Revised by Arita and Gorbunov (2000), they were transferred to the genus *Pyrophleps*. The remaining four species, mostly caught with the use of synthetic pheromones, were described fairly recently: *P. nigripennis* Arita & Gorbunov, 2000, *P. vitripennis* Arita & Gorbunov, 2000, *P. cucphuonganae* Arita & Gorbunov, 2000 and *P. bicella* Xu & Arita, 2015. Thus, nothing was known until now about the behaviour of members of this genus. An identification key to the species of *Pyrophleps* has been published by Xu et al. (2015).

A wasp-mimicking sesiid was observed and collected in lowland dipterocarp forests of Peninsular Malaysia. Its flight and mud-puddling behaviour, as well as functional morphology, are described and shown in a video. Time and conditions of occurrence are provided. Based on morphological analyses as well as DNA barcode, it is described here as a new species, which leads the genus *Pyrophleps* to now count eight species.

## Materials and methods

The behaviour of the new species was observed and filmed in its habitat. Using an electronic thermo-hygrometer placed in the shade, temperature and air humidity were measured. Two specimens were collected near Merapoh, Pahang, Malaysia without the use of synthetic pheromones. A further three individuals were observed and photographed but were not collected (the species was observed in total seven times; however, as three observations were made three days in a row, they could relate to the same individual, and only three were photographed in the wild). Morphological details were studied with a Leica M80 stereomicroscope and photographed using a Leica M205A. Wingspan, body and antenna length were measured on a computer screen from photographs of mounted specimens taken next to a scale. Genitalia were prepared by maceration of the abdomen in boiling 10% KOH, dissection in 10% ethanol, and pieces dehydrated by passing through 30%, 60%, and 100% ethanol and mounted in Euparal. DNA barcoding was conducted on total DNA isolated from a single leg of the paratype in the Canadian Centre for DNA Barcoding, University of Ontario, Guelph, Canada following Ivanova et al. (2006). The barcode sequence (available in the Barcode of Life Database, BIN number: BOLD:ACS2287) was analyzed through the Basic Local Alignment Search Tool (BLAST), Barcode of Life Data System Identification tool (Ratnasingham and Hebert 2007), CLC Sequence Viewer and SnapGene 3.3.4. Barcodes for comparisons were taken from BOLD.

## Results

### Pyrophleps ellawi

Taxon classificationAnimaliaLepidopteraSesiidae

Skowron Volponi sp. n.

http://zoobank.org/78854E0D-F0CE-4515-9581-4E06959411A5

Figs 1 , 2 , 3 , 4

#### Type material.

Holotype ♂ (Fig. 3): “Malaysia: Pahang, Merapoh, 04°39.04'N, 102°01.80'E, 21 III 2017, Skowron Volponi M.A.” / “Holotype, *Pyrophleps ellawi* sp. n., des. Skowron Volponi M.A. 2017”. Paratype ♂: “Malaysia: Pahang, Kuala Tahan, 04°22.98'N 102°23.98'E, 07 VIII 2014, Skowron Volponi M.A.” / “Paratype, *Pyrophleps ellawi* sp. n., des. Skowron Volponi M.A. 2017”. In coll. Marta Skowron Volponi (Gdansk).

#### Description.

Alar expanse: 16–19.5 mm. Body length: 9.5–12 mm.

Head: antenna 6–6.5 mm, clavate, black dorsally, admixture of brick orange scales ventrally, several pale yellow or white scales at base, acuminate seta at apex; frons smoothly scaled, black with silver sheen; vertex covered with elongated, hair-like scales, bright orange mixed with pale yellow and several black scales between ocelli; smooth white scales adjacent to compound eye; ocelli brown; eyes red; proboscis orange, well-developed, functional; labial palpus long, upturned, with elongated black, orange and white scales dorsally and apically, shorter white scales ventrally; pericephalic hairs white with several orange ones dorsally, black ventrally.

**Figure 1.**
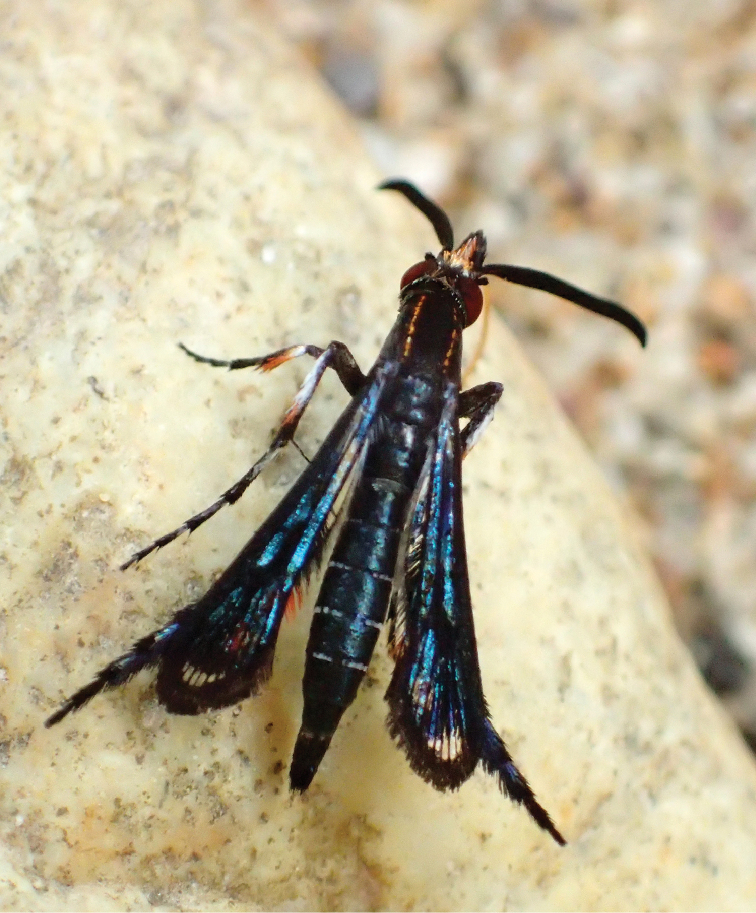
*Pyrophleps ellawi* has a strong blue sheen in sunlight. Representatives of this species vary in the number of orange scales on the thorax. The scales form two longitudinal stripes, either dashed or solid.

**Figure 2.**
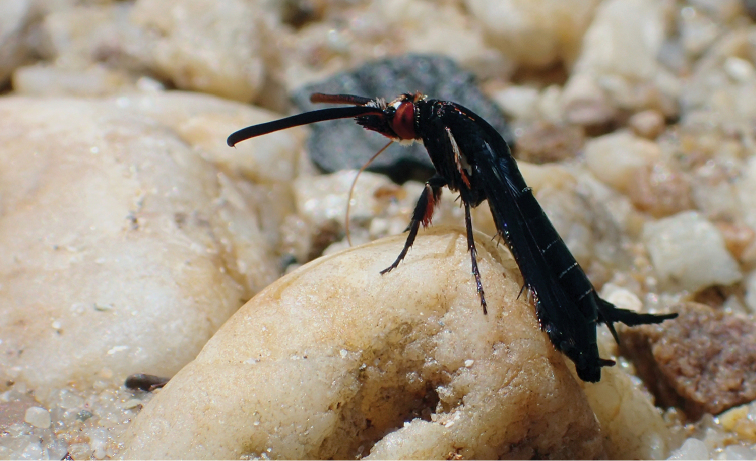
*Pyrophleps ellawi* puddling on a river bank. Note the curled-up hind leg tarsi.

Thorax: smoothly scaled, black with blue sheen, narrow longitudinal orange stripes (solid or dashed) dorsally; patch of white and individual orange scales laterally; elongated hair-like scales at wing insertion, white mixed with black; patagia black. Legs: fore coxa white with several orange scales ventrally; fore- and mid-femora smoothly scaled, black; fore tibia orange dorsally and black ventrally, tufted with hair-like scales (Fig. 2), smooth white scales dorsally at base; 1^st^ tarsomere black with orange and yellow scales basally, tarsomeres 2–3 black with white scales dorsally at base, tarsomeres 4–5 black; mid tibia smooth-scaled ventrally, black with admixture of white scales, dorsally elongated scales white basally, orange medially and black distally; spurs black away from body and white towards body; tarsomeres black dorsally and white proximally but black distally on ventral side; hind femur black; hind tibia smooth-scaled, black in proximal half, tuft of hair-like scales covering distal half of tibia away from body and towards body and 1^st^ and 2^nd^ tarsomere only towards body: tibia with outer scale vestiture black with admixture of white, inner scale vestiture black and brick orange, on 1^st^ tarsomere orange and black, on 2^nd^ tarsomere shorter black scales; tarsomeres black with several white scales at base of 1^st^ and 2^nd^ tarsomere or only 1^st^. Spurs black away from body, white towards body. All legs with metallic sheen in sunlight. Forewing: hyaline; transparent areas covered with semi-hyaline scales with strong blue sheen (Fig. 1); veins, margins and fringe black; discal spot broad, black, with black extensions into anterior transparent area (ATA) along discal cell boundary and into external transparent area (ETA) between middle of cell M2-M3 and vein R3. ETA divided into nine cells, of which the two between veins R4-R3 and M3-CuA1 are additionally divided by narrow longitudinal stripes. Hindwing: transparent with semi-hyaline scales with blue sheen in distal half between veins 1A and M1, and at base. Discal spot narrow.

Abdomen: black with blue sheen, brighter strongly light-reflecting bands on margin of each tergite; admixture of white scales ventrally; anal tuft very small, black. Male genitalia (Fig. 4): tegumen broad proximally, gradually tapered towards uncus; saccus short with broad, slightly bifurcate base; valva broadening from 1/3 length, margins densely covered with long setae, sparser hair-like setae medially; uncus with ring of brown setae; gnathos narrow, long, pointed distally; aedeagus about 1.5 length of valva.

**Figure 3.**
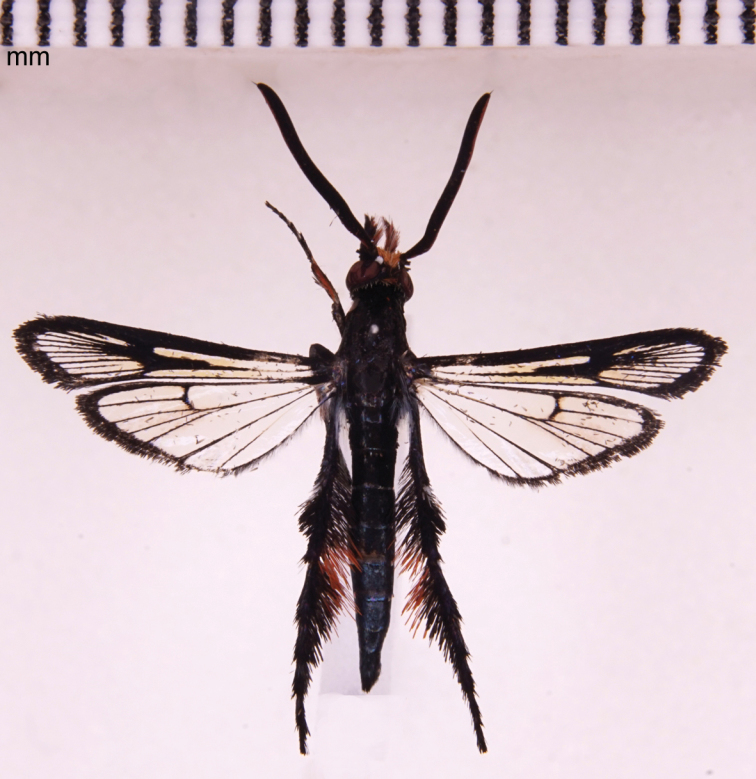
Male holotype of *Pyrophleps ellawi*.

**Figure 4.**
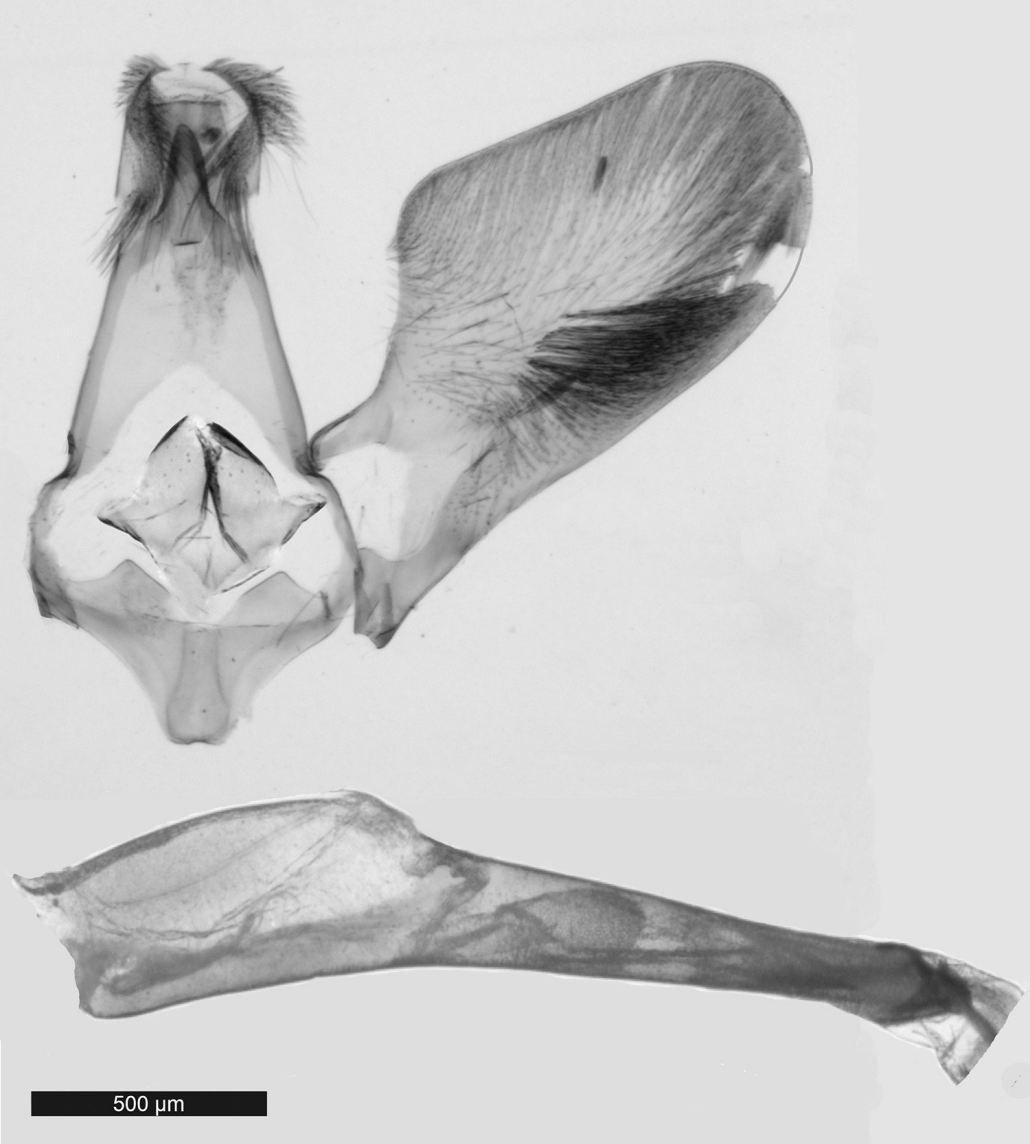
Male genitalia of *Pyrophleps ellawi*.

#### Variability.

The new species varies in the number of orange scales on thorax which form two, either dashed or solid, longitudinal stripes (Figs 1–2, Suppl. material 1 video TC: 00:40–00:57). On the hind legs white scales are present on the 2^nd^ tarsomere in the holotype, in the paratype 2^nd^ tarsomere is entirely black. It also differs in size.

#### Diagnosis.

The new species is superficially most similar to *Pyrophleps vitripennis*, from which it can easily be distinguished by the configuration of male genitalia (compare Fig. 4 herein with Arita and Gorbunov 2000, fig. 6), presence of hair-like scales on labial palpi (smoothly scaled in species compared), presence of orange hair-like scales on fore- and mid tibiae and lack of orange scales on wings, broader external transparent area of forewing. Apart from morphological features, *P. ellawi* shows 8% COI sequence divergence from *P. vitripennis* (Table 1). Based on genitalia configuration, this species is most similar to *P. nigripennis*. However, it differs in the shape of the valva and gnathos. Besides that, it can immediately be distinguished by the well-developed transparent areas of forewing (compare Fig. 3 herein with Arita and Gorbunov 2000, fig. 8), narrow discal spot on hindwing and by the colouration of the hind leg tuft (extensive red both externally, on tibia, and internally, on tibiae and tarsi, in *P. nigripennis*). From *P. ruficrista* it differs in more developed forewing ATA and PTA and less developed ETA and in the hind leg tuft (cinnabar red with two black spots and patches of blue scales in *P. ruficrista*). From *P. cruentata*, *P. haematochrodes* , *P. cucphuonganae* and *P. bicella*, it can be distinguished by the entirely transparent hindwings and absence of red scales on wings and abdomen.

**Table 1.** COI pairwise sequence divergence of species closely related to *Pyrophleps ellawi* with Barcode of Life BIN numbers. Multiple alignment of the compared sequences is shown in Supplementary material 2.

Species	BIN number	Pairwise sequence divergence from *Pyrophleps ellawi*
*Pyrophleps vitripennis*	BOLD:ABX4445	7.90%
*Heterosphecia pahangensis*	BOLD:ACJ6445	10.03%
	BOLD:ACV6125	9,68%
*Heterosphecia bantanakai*	BOLD:ABU6338	9.88%
*Heterosphecia tawonoides*	BOLD:ACJ6387	11.70%
*Aschistophleps longipoda*	BOLD:ABW9181	11.09 %

#### Etymology.

The species is named after our dear friend El Law, a dedicated conservation activist with sincere sensibility for Malaysian nature who, over the years of our studies on Malaysian Sesiidae, offered us his help in countless aspects.

#### Distribution and habitat.

**Figure 5.**
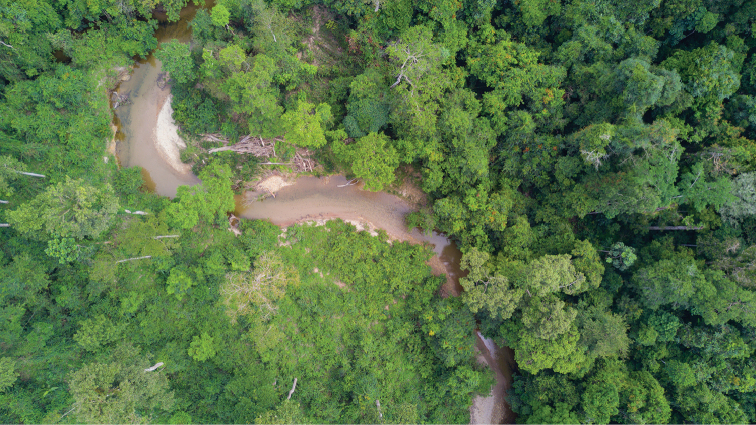
Habitat of *Pyrophleps ellawi*: sandy/pebble river banks in a lowland dipterocarp forest, Malaysia.

In addition to the type locality, the species is known also from the Taman Negara National Park, Malaysia, where it was observed and filmed in two locations approx. 50 km from each other. All observations were done on sandy and pebble river banks exposed to sunlight, in a lowland dipterocarp forest (Fig. 5)

#### Behaviour.

*Pyrophleps ellawi* was observed flying around sandy and pebble beaches on a river bank and stopping now and again to mud-puddle (Suppl. material 1 video TC: 00:12–00:32). The flight is rapid, very similar to that of Eumeninae wasps. Many individuals of the wasp *Coeleumenes burmanicus* Bingham, 1897 were seen puddling in the same area. When flying, the wasp and the sesiid were impossible to distinguish. Both the flight path and velocity were very similar. When on land, *P. ellawi* moved around frantically, searching for moisture with its long proboscis (Suppl. material 1 video TC: 00:33–01:04). It usually landed for a moment only and never stayed for more than a few minutes on the same beach. When puddling, it keeps its wings folded back (Figs 1–2) and uses its fore and mid legs for locomotion (Suppl. material 1 video). The long hind legs do not seem to be fully functional in terms of locomotion. *Pyrophleps ellawi* keeps the hind tarsi curled upwards (Fig. 2) and occasionally it makes flapping movements which sometimes end in tapping the ground (Suppl. material 1 video TC: 00:48–00:54; 01:05–01:24). *Pyrophleps ellawi* was first seen in August 2014 (one observation), then in May 2016 (three observations 2 and 5 days apart) and in March 2017 app. one week after an extended period of heavy rains associated with the Northeast Monsoon (3 observations 3 days in a row and 1 in a different location approx. 50 km away). It flies in the afternoon, between 1:30 and 4:00 pm with temperature 30–32°C and air humidity 60–80%. Each observation was of a single individual.

**Figure 6.**
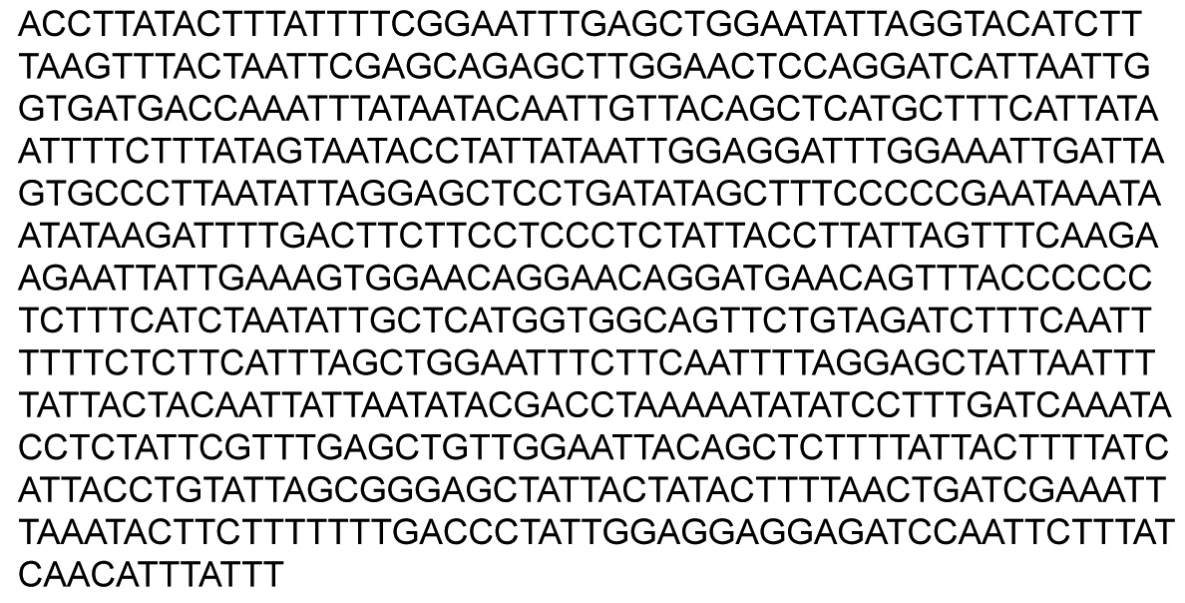
658-bp DNA sequence of the mitochondrial cytochrome c oxidase subunit I gene of *Pyrophleps ellawi* paratype. Barcode of Life BIN (Bold Identification Number): BOLD:ACS2287.

## Discussion

*Pyrophleps ellawi* is associated with river banks in primary rainforests of Peninsular Malaysia. It was repeatedly observed mud-puddling, in the same location as *Heterosphecia pahangensis* Skowron, 2015 and *H. tawonoides* Kallies, 2003 (personal observations). In a different location, the authors also observed *P. cruentata* and *P. ruficrista* puddling on a river bank. Taking this into account, as well as the fact that Le Cerf (1912) collected *P. haematochrodes* near a river in Vietnam, it is possible that puddling on river banks is a typical behaviour of representatives of this genus, and perhaps of other Oriental Sesiidae.

Members of the family Sesiidae are very rarely filmed in their habitats. Most often, only their morphology is described in detail. This is also the case in Oriental Sesiidae, including the genus *Pyrophleps*; nearly nothing is known about their behaviour. Observations of sesiids in the wild provide priceless information on their biology and even on their true posture, which is lost once the insects are pinned. The video included in this publication allowed the authors to note the intense blue sheen of the sesiid in sunlight, its natural resting position, the functionality of its legs and mud-puddling behaviour, aspects entirely unknown for other species of *Pyrophleps*. Thus, we encourage other entomologists to film sesiids instead of collecting them straight away.

Arita and Gorbunov (2000) state that species of *Pyrophleps* fly from April to July, and in September, and suggest they have two generations per year. *Pyrophleps ellawi* was seen in March, May and August. *H. pahangensis* and *H. tawonoides* also occurred in this period. The authors of this work monitored the location also in April, June and July and although *H. pahangensis* and *H. tawonoides* were seen repeatedly during this time, *P. ellawi* was absent. This indicates it has a different life cycle than the observed *Heterosphecia*, and might have three generations per year. However, there might as well be additional generations in periods which we did not study. Moreover, voltinism may differ from year to year depending on the amount of rainfall and subsequent dry periods, associated with both monsoons and variable El Niño events affecting Malaysia (Corlett and Primack 2011). Due to the complex climate of Malaysia and also based on our observations, caution is needed when making conclusions about the number of generations Sundanian species of Sesiidae have per year, e.g. *P. ellawi* or *P. ruficrista*. *Pyrophleps ellawi* appeared after only several days of sunshine and high temperature following a long period of rain. This may mean that a rise of temperature and end of rain trigger emergence of *Pyrophleps* (perhaps after an extended period spent as a larvae or pupae during the monsoon). Other factors, such as the moon phase, may also influence timing of emergence, which would be an interesting topic for future studies. It is also worth noting that all observed individuals seemed to have freshly emerged.

Although *P. ellawi* occurs in the same locations as *H. pahangensis* and *H. tawonoides*, which are known to be bee mimics in both morphology and behaviour (Skowron et al. 2015; Skowron Volponi and Volponi 2017), and shows similar mud-puddling behaviour, it flies in a completely different manner and cannot be confused with these two species. However, in the field, it had been repeatedly confused with Eumeninae (potter) wasps, whose rapid flight it closely resembles. Adding to this the slender body, long legs (with hind leg tuft much smaller than in *Heterosphecia*, barely visible from underneath the wings when the sesiid perches, thus unlike the pollen-laden hind legs of bees) and strong blue sheen of *P. ellawi* (Figs 1–2), it seems that the new species is a mimic of Eumeninae wasps.

## Conclusion

The new wasp-mimicking species of Sesiidae, *Pyrophleps ellawi*, represents the first record of the genus *Pyrophleps* in Peninsular Malaysia and the first filmed in the wild. The video realized in its habitat provided valuable information on its authentic habitus, functional morphology, and behaviour.

## Supplementary Material

XML Treatment for Pyrophleps ellawi

## Supplementary materials

Supplementary material 1 Video of *Pyrophleps* ellawi in natural habitat Data type: multimedia Explanation note: Supplementary video from the wild shows the behaviour and authentic postures of *Pyrophleps ellawi*. High resolution video available from Vimeo on: https://vimeo.com/230445159 This dataset is made available under the Open Dafigase License (http://opendatacommons.org/licenses/odbl/1.0/). The Open Dafigase License (ODbL) is a license agreement intended to allow users to freely share, modify, and use this Dataset while maintaining this same freedom for others, provided that the original source and author(s) are credited.Marta Agnieszka Skowron Volponi, Paolo Volponi

Supplementary material 2 Multiple alignment of barcode sequences Data type: mollecular data Explanation note: Multiple alignment of barcode sequences of the following species: *Pyrophleps ellawi*, *P. vitripennis*, *Heterosphecia pahangensis* (3 specimens), *H. bantanakai*, *H. tawonoides*, *Aschistophleps longipoda*. This dataset is made available under the Open Dafigase License (http://opendatacommons.org/licenses/odbl/1.0/). The Open Dafigase License (ODbL) is a license agreement intended to allow users to freely share, modify, and use this Dataset while maintaining this same freedom for others, provided that the original source and author(s) are credited.Marta Agnieszka Skowron Volponi, Paolo Volponi

## References

[B1] Arita Y Gorbunov O (2000) Notes on the tribe Osminiini (Lepidoptera, Sesiidae) from Vietnam, with descriptions of new taxa. Transactions of the Lepidopterological Society of Japan 51(1): 49–74.

[B2] Corlett RT Primack RB (2011) Tropical Rain Forests: An Ecological and Biogeographical Comparison. Second edition. Wiley-Blackwell, West Sussex, UK, 9–13. https://doi.org/10.1002/9781444392296

[B3] Ivanova NV de Waard JR Hebert PDN (2006) An inexpensive, automation friendly protocol for recovering high-quality DNA. Molecular Ecology Note 6: 998–1002. https://doi.org/10.1111/j.1471-8286.2006.01428.x

[B4] Le Cerf F (1912) Description de deux Aegeriidae nouvelles [Lep.]. Bulletin de la Société entomologique de France 1912: 54–55.

[B5] Ratnasingham S Hebert PDN (2007) Barcoding. BOLD: The barcode of life data system (www.barcodinglife.org). Molecular Ecology Notes 7(3): 355–364. https://doi.org/10.1111/j.1471-8286.2007.01678.x 18784790 10.1111/j.1471-8286.2007.01678.x PMC1890991

[B6] Skowron MA Munisamy B Binti Ab. Hamid S Węgrzyn G (2015) A new species of clearwing moth (Lepidoptera: Sesiidae: Osminiini) from Peninsular Malaysia, exhibiting bee-like morphology and behavior. Zootaxa 4032(4): 426–434. https://doi.org/10.11646/zootaxa.4032.4.7 26624378 10.11646/zootaxa.4032.4.7

[B7] Skowron Volponi MA Volponi P (2017) A 130-year-old specimen brought back to life: a lost species of bee-mimicking clearwing moth (Lepidoptera: Sesiidae: Osminiini) rediscovered in Peninsular Malaysia’s primary rainforest. [Submitted]

[B8] Xu HM Arita Y Chen B Wang M (2015) Description of *Pyrophleps bicella* (Lepidoptera: Sesiidae), a new Chinese species of clearwing moth. Florida Entomologist 98(1): 149–151. https://doi.org/10.1653/024.098.0125

