# Supplementary figures and images for: A new species of wasp-mimicking clearwing moth from Peninsular Malaysia with DNA barcode and behavioural notes (Lepidoptera, Sesiidae)
Source: Zookeys. 2017 Aug 21;(692):129–39. doi: 10.3897/zookeys.692.13587 (PMC5672733; doi:10.3897/zookeys.692.13587)

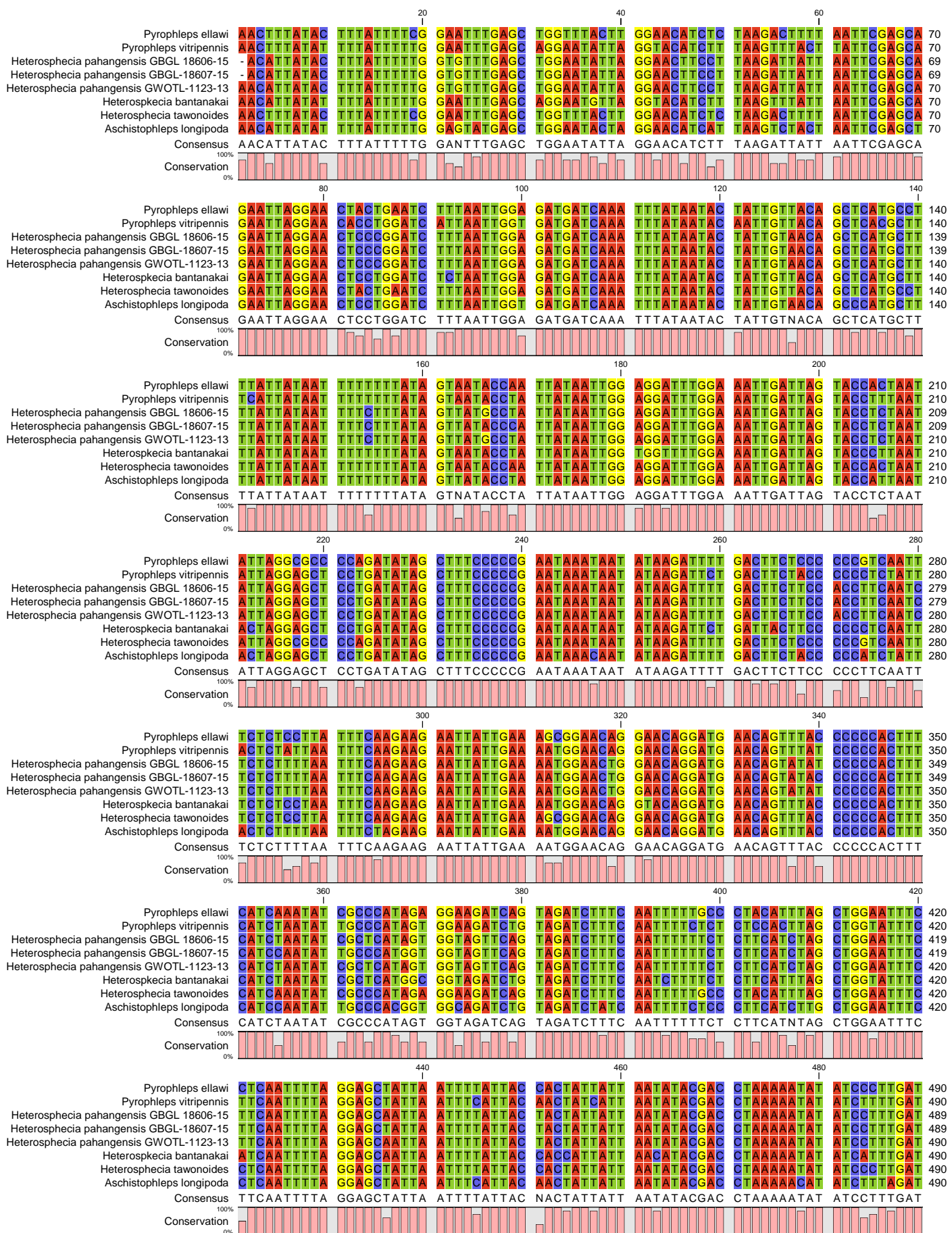

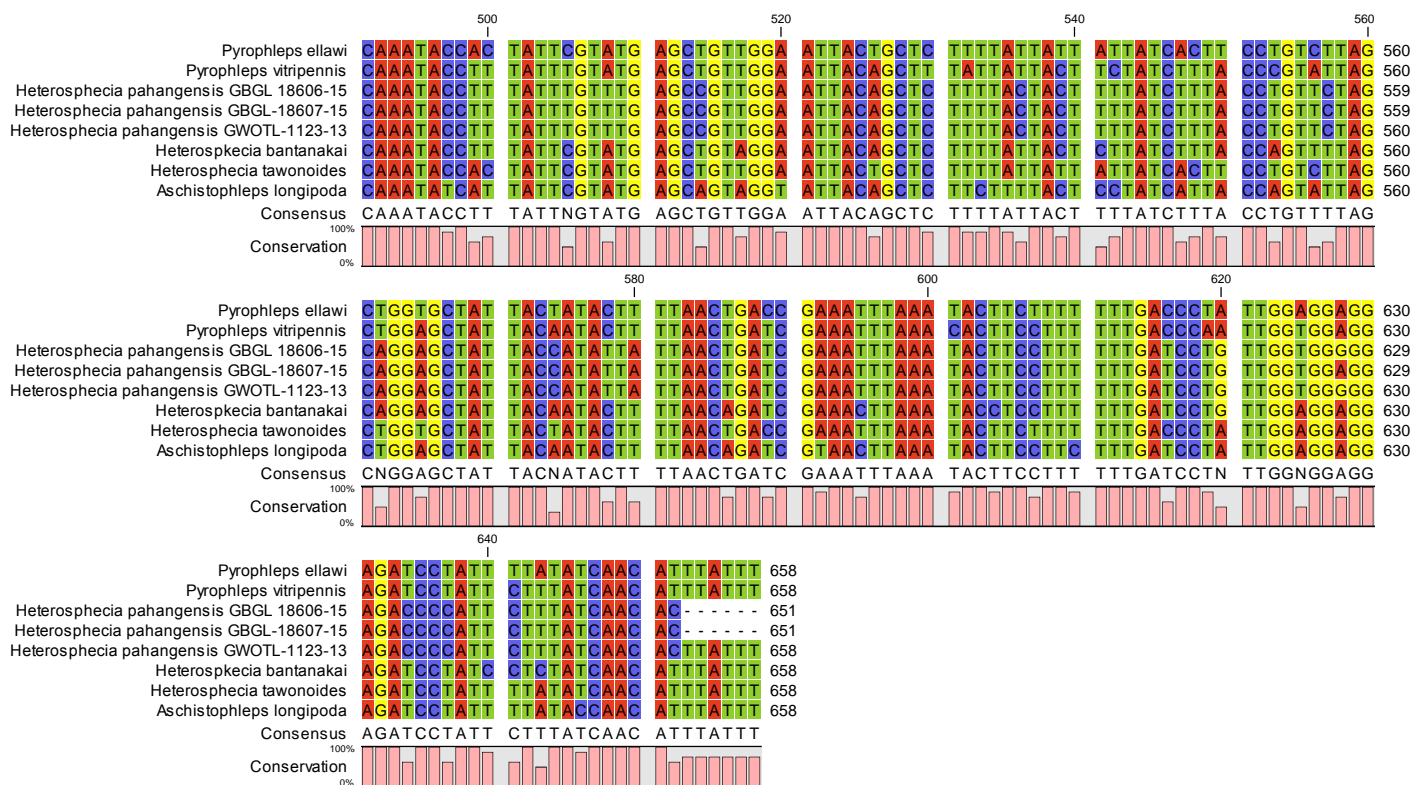

Supplement: Supplementary material 2 — Multiple alignment of barcode sequences [file zookeys-692-129-s002.pdf]
